# A discovery and development roadmap for new endectocidal transmission-blocking agents in malaria

**DOI:** 10.1186/s12936-018-2598-5

**Published:** 2018-12-10

**Authors:** Jeremy Burrows, Hannah Slater, Fiona Macintyre, Sarah Rees, Anna Thomas, Fredros Okumu, Rob Hooft van Huijsduijnen, Stephan Duparc, Timothy N. C. Wells

**Affiliations:** 1 Medicines for Malaria Venture, Route de Pré Bois 20, 1215 Geneva 15, Switzerland; 2 PATH, 2201 Westlake Avenue, Seattle, WA 98121 USA; 3 Department of Infectious Disease Epidemiology, MRC Centre for Global Disease Analysis, Imperial College London, Norfolk Place, London, W2 1PG UK; 4 Innovative Vector Control Consortium, Liverpool School of Tropical Medicine, Pembroke Place, Liverpool, L3 5QA UK; 5 Environmental Health and Ecological Sciences Department, Ifakara Health Institute, Off Mlabani Passage, Ifakara, Morogoro United Republic of Tanzania; 6 School of Public Health, Faculty of Health Sciences, University of the Witwatersrand, Parktown, Republic of South Africa; 7 Institute of Biodiversity, Animal Health and Comparative Medicine, University of Glasgow, Glasgow, UK

**Keywords:** Malaria, *Plasmodium*, Transmission blocking, Endectocides, target candidate profile, Target product profile, Transmission reducing agent

## Abstract

Reaching the overall goal of eliminating malaria requires halting disease transmission. One approach to blocking transmission is to prevent passage of the parasite to a mosquito, by preventing formation or transmission of gametocytes. An alternative approach, pioneered in the veterinary field, is to use endectocides, which are molecules that render vertebrate blood meals toxic for the mosquito vector, also killing the parasite. Field studies and modelling suggest that reducing the lifespan of the mosquito may significantly reduce transmission, given the lengthy maturation process of the parasite. To guide the development of new endectocides, or the reformulation of existing molecules, it is important to construct a framework of the required attributes, commonly called the target candidate profile. Here, using a combination of insights from current endectocides, mathematical models of the malaria transmission dynamics, and known impacts of vector control, a target candidate profile (TCP-6) and a regulatory strategy are proposed for a transmission reducing agent. The parameters chosen can be used to assess the potential of a new medicine, independent of whether it has classical endectocide activity, reduces the insect and parasite lifespan or any combination of all three, thereby constituting an ‘endectocidal transmission blocking’ paradigm.

## Background

### Eradicating malaria: thinking about preventing transmission in addition to treatment options

Malaria remains one of the leading causes of morbidity and mortality, with an estimated 435,000 deaths in 2017, 93% of which were in Africa [1]. Between 2010 and 2016, the incidence of malaria infection decreased by 18%. This prevention of infection was largely driven by the deployment of insecticide-treated nets (ITNs), indoor residual spraying (IRS), and the introduction of high quality treatments, which show a significant ‘post-treatment prophylaxis’ [2]. Recently, there has also been a dramatic increase in the deployment of medicines to protect vulnerable populations, including seasonal malaria chemoprevention (SMC) for children under 5 years of age [3, 4] and intermittent preventive treatment in pregnancy (IPTp, [5]). Over the same period (2010–2016), there has been an even larger decrease in the mortality rate, 32% [1]. This difference points to increased survival of infected individuals, which can be attributed to better case management. Key factors here are the more widespread use of diagnostics, wider availability of high-quality, fixed-dose artemisinin combination therapy (ACT, [6]), and the switch to injectable artesunate treatment of severe malaria. In 2015, the World Health Organization (WHO) announced its global technical strategy for malaria for 2016–2030, which sets an ambitious target of reducing malaria incidence and mortality by a further 90% [7]. This strategy has two major pillars, the first of which is to ensure universal access to malaria prevention, diagnosis and treatment, and the second, to accelerate efforts towards malaria elimination. To achieve this, it will be important to continue the investments to develop new generations of all the interventions described above, particularly for vector control and case management.

### Basic concepts of malaria transmission control

The theoretical analysis of disease eradication can be summarized as the need to reduce the number of secondary infections coming from an initial infection in a fully susceptible population, to the extent that transmission is eventually halted. This is described as the basic reproduction number, R_0_ [8]. Only by reducing R_0_ to < 1 over extended transmission cycles can local elimination be achieved. In its simplest early form, the basic reproduction rate is defined by six parameters in the following equation:

$$ R_{0} = \frac{{a^{2} bcm}}{r\mu } $$

Three parameters relate to the mosquito vector: *m*, the number of mosquitoes per human host; 1/*µ*, the life expectancy of the mosquitoes (*µ* is a measure of daily mortality); and *a*, the rate of biting humans. Two parameters describe the life cycle in the human host: *b*, the transmission efficiency from mosquitoes to human; and 1/*r*, the duration of the disease in humans (*r* is a measure of recovery rate of infected people). Finally, two parameters describe the infection of mosquitoes by humans: *c*, the transmission efficiency from human to mosquito, and once again *a*, the rate of the mosquitoes biting humans.

One approach to transmission blocking is ITNs. Fertilized female mosquitoes of the genus *Anopheles* require a blood meal to successfully produce eggs, and typically seek out humans between dusk and dawn. ITNs provide a lethal barrier, preventing mosquitoes from biting humans and killing the insects [9]. Insecticides used in today’s ITNs mostly belong to the pyrethroid class [10], although second-generation nets under consideration include those with additional actives, such as the pyrrole, chlorfenapyr [11], the synergist, piperonil-butoxide [12], or the insect growth regulator, pyriproxyfen [13]. By preventing biting, these nets ensure blood-feeding inhibition (BFI) in addition to killing the mosquitoes, where the vectors are still susceptible. Another approach is IRS. Blood-fed mosquitoes typically rest on indoor wall surfaces, during which time they release excess liquid and regain full flight capacity, or stay longer to digest their blood meals to gravid status. Unfed mosquitoes may also temporarily rest on the wall surfaces. These resting mosquitoes can be targeted to different degrees by IRS. Both ITNs and IRS therefore lower the number of mosquitoes, *m*, the rate of biting *a*, but most importantly the daily survival probabilities and therefore life expectancy of the mosquitoes, 1/*µ*. However, resistance has now been observed to all the classes of insecticides used both in ITNs and IRS [14], meaning that the full value of these interventions is heavily compromised. In such cases, pyrethroid-treated ITNs may still provide a physical barrier (reducing the biting rate) and limited toxicity, even among resistant insects, so at least the personal protection is retained even when the communal benefits usually associated with the killing effect of nets are lost. However, resistance heavily compromises the protective value of IRS [15], and there has been a significant decline in the use of IRS over recent years. In addition to the evolution of insecticide resistance, mosquito populations in Africa are changing their behaviour to avoid the indoor environment [16–19], with an increasingly significant proportion of biting now occurring outdoors and early in the evenings [20–23].

New approaches are therefore urgently needed to complement ITNs and IRS for vector control. Attractive targeted sugar baits (ATSB) are one approach where an insecticide is incorporated in a membrane-bound sugar bait to attract and kill mosquitoes, typically placed just outside houses, thus reducing transmission at the population level [24]. An alternative approach is the gene-drive approach, where genetically engineered insects carry a sterility gene, and spread it to the population [25], or where the *Anopheles* are engineered to become refractory to *Plasmodium* infections [26]. Multiple other options, with different levels of evidence for success, have been proposed, including expansion of larval source management, use of area-wide spatial repellents either alone or in combination with traps to form push–pull systems, improved housing, use of entomopathogenic fungi, and use of endectocides among others (reviewed in [27]).

### Current case management of malaria patients may leave significant gametocytaemia

Clinically, transmission can be reduced either by decreasing the number of gametocytes in a patient, or decreasing their ability to be transmitted, thus lowering the proportion of infective bites, *b*. Existing anti-malarial treatments differ significantly in their capacity to reduce gametocyte carriage, because they were primarily developed to kill blood-stage parasites, and gametocytes produce no symptoms. A recent meta-analysis of 121 trials found that the prevalence of gametocyte-carrying patients dropped six-fold after ACT [28], but there was no association between the rate of asexual parasite clearance and gametocytaemia during follow-up. All new pre-clinical development candidates in the global malaria portfolio (http://www.mmv.org; [29]) are now routinely tested for their transmission-blocking activity as assessed by standard membrane feeding assays (SMFAs; [30]), but case management is unlikely to greatly impact malaria transmission for the moment.

### Endectocides used in transmission blocking

A remaining possibility is the idea of giving a drug to an infected individual that results in the death of the arthropods feeding on them. Endectocides have been commonly used in veterinary practice to reduce or eliminate ticks in companion animals, such as *Ctenocephalides felis* in cats and multiple species of ticks (*Rhipicephalus sanguineus*, *Ixodes ricinus*, *Dermacentor reticulatus*) in dogs. These treatments are mainly aimed at the parasites that these ticks transmit. Lyme disease in humans is the result of infection by the tick-borne spirochete *Borellia burgdorferi*, and many more human tick-borne diseases have been described [31]. Although Lyme disease is typically treated by 14–21 days of antibiotic treatment, early administration of an endectocide, ivermectin, could be beneficial in some settings. Studies with fluralaner [32] in dogs have shown 100% mortality of the tick within 12 h after oral treatment, which would be sufficiently fast to potentially prevent the primary infection by Lyme disease. A recent study has also highlighted the potential for repurposing the isoxazolines, fluralaner and afoxalaner to kill mosquito vectors for malaria and dengue [33]. In human medicine, ivermectin is used to kill the parasites in patients infected with the mite *Sarcoptes scabiei*, which results in scabies [34].

When the parasites that vectors spread are the more relevant target, rather than the vectors themselves, the relation between drug exposure and how this shortens the lifespan of the insect becomes particularly relevant for malaria transmission [35–39]. Even under optimal climatic situations, *Plasmodium falciparum* requires 10–12 days for its gametes, once taken up by mosquitoes, to produce infectious sporozoites [40]. The natural lifespan of the mosquito is typically 1–2 weeks, so shortening the lifespan of the insect may prevent the formation of infectious sporozoites, and provide powerful transmission-blocking opportunities. Clearly, the transmission-blocking activities of new drugs in the insect stages includes several options for shortening the host and parasite lifespan, and moves the discussion beyond the classical definition of an endectocide. It would seem easier to use medicines that ‘merely’ prevent the differentiation of gametocytes into sporozoites, as opposed to those that kill the entire insect; however there are only examples of the latter, and drugs that specifically target sporozoite development are very difficult to discover, so this review focuses on the classical endectocides, although the new TCP-6 covers additional scenarios.

### The experience of ivermectin use in preventing malaria transmission

Over recent years there has been growing interest in the use of ivermectin, as a potential endectocide, for a use in malaria. Several clinical studies have been conducted, [35, 41–45], and the WHO has published a Meeting Report on Ivermectin for malaria transmission control [46]. The definition of the Target Product Profile does depend on the proposed deployment; and this report focused on the use case for high-dose ivermectin as a stand-alone mass drug administration (MDA) regimen. This initial research has suggested that the relatively short human plasma half-life of the molecule (approximately 12–36 h) may require a much higher dose or more frequent administration than the standard single 150–200 μg/kg used in helminth control, or long-acting formulations, such as recently demonstrated in Tanzania, where efficacies in cattle lasted over 6 months [47]. The alternative approach would be to use ivermectin in combination with other malaria control modalities. In another example from Tanzania, addition of ivermectin to long-lasting, insecticidal-treated bed nets (LLINs) resulted in a near-complete collapse of populations of the malaria vector, *Anopheles arabiensis*, inside large naturalized mesocosms [48]. Combining ivermectin with SMC is another option, where 3 days of ivermectin could be given each month with a full treatment course of anti-malarials [49, 50]. This also underscores an additional risk in use of endectocides in malaria elimination; the possibility that insects could emerge which are no longer sensitive to ivermectin, as is seen for other targeted arthropods [51] and helminths [52]. In this case, it is always important to consider these endectocides as complementary to other vector control interventions (such as LLINs or IRS) that use insecticides that remain effective even against ivermectin-resistant mosquitoes.

### A target candidate profile for endectocidal transmission blocking in malaria

This paper explores the use case for an ideal medicine for blocking transmission through endectocidal activity. This requires a definition of the ‘use case’, the way such a medicine would be deployed, and several are in discussion: MDA as a stand-alone therapy, inclusion with SMC regimens, or in addition to treatment regimens, all three use cases being complementary to core vector control interventions such as ITNs or IRS. The requirement for such uses would be described in a target candidate profile (TCP) that includes comments about formulations, and combinations required for the ideal product.

TCPs have been developed for other types of anti-malarial medicine, and help define the screening cascade, and the supportive data required [53]. Profiles for compounds which address blood-stage asexual parasites (TCP-1), relapse of the dormant hypnozoites (TCP-3), hepatic schizonts (TCP-4), and transmission blocking via gametocytes (TCP-5) have been previously defined [53]. In this review, a new target candidate profile, TCP-6, is described for an endectocide, where the reference molecule is currently elevated-dose ivermectin. The definition of the endectocide in this case is expanded to include existing examples that kill blood-feeding *Anopheles*, and the concept that it is actually also a transmission-reducing and transmission-blocking agent. Following the development of the TCP, there is a discussion of the development and regulatory pathway. Even for molecules that are currently on the cusp of preclinical development, such as the isoxazolines, regulatory approval for use in adults and children may take 8–10 years. Given these very long time-horizons, it is essential to have a clear vision of how to proceed, and a widely agreed framework, but with the understanding that new evidence may necessitate adjustments of such frameworks and pathways. The proposal of the endectocidal transmission-blocking molecule TCP-6 is made in full knowledge that this will be modified in the light of new data.

### General outline of the TCP-6 profile

When considering the features proposed in a new TCP-6 (outlined in Table 1) it is important consider the use case. Compounds developed for TCP-6 use in combination with SMC are ideally required to still be present in human blood at day 28 after administration at a concentration sufficient to decrease the mean lifespan of malaria-competent female *Anopheles* that take a blood meal, since this is also the administration period for SMC. This, and the requirement to minimize the dose size requires that the intrinsic potency of a new TCP-6 molecule should be high, with, ideally, clear activity in the nanomolar range. Poor potency may result in high dosing (> 10 mg/kg), difficulties in co-formulation with other anti-malarials, problems when formulating fixed-dose combinations, and high cost of goods. Therefore, during the lead optimization work, the optimization of potency and pharmacokinetics will need special attention. Second, a faster-acting compound is preferred, which will be linked with the mechanism of action and the mosquito tissue distribution. There is a risk of transmission in a malaria-endemic region with every blood meal. Any compound that is long-lasting in the vertebrate host but is able to dramatically decrease mosquito survival to fewer than 10 days post mosquito infection (the estimated extrinsic incubation period of *Plasmodium*) will be particularly effective as they would stop pathogen development in the mosquito-midgut [40]. Any compound able to kill rapidly and thus reduce egg laying, as already demonstrated with some formulations of ivermectin when administered to cattle [43] and humans [48], will also greatly impact the vector population. Third, since a new compound with pure TCP-6 activity targets the mosquito rather than the human, and the initial use case involves children, the safety and tolerability profile need to be extremely convincing, with a wide safety margin. There are, in fact, safety concerns with ivermectin [54]. An alternative is to consider these endectocide transmission-blocking candidates primarily for use in adults, in which case communal benefits are still accruable even by children.

**Table 1:** target candidate profile for a new endectocide

TCP-6 criteria at human proof of concept	Minimum essential	Ideal
Dosing regimen	Oral, once monthly three daily doses of < 10 mg/kg	Oral, once monthly single dose < 2 mg/kg
Action and clinical parasite reduction ratio from single dose	Efficacy of a Hazard ratio at least 4 is delivered upon mosquito feeding 28 days post oral dose (for a use with SMC)	Rapid onset of action, within 12 h. Efficacy equal or higher than a hazard ratio of 4 is delivered upon mosquito feeding 30 days post oral dose
Susceptibility to loss of efficacy due to acquired resistance in mosquitoes	No fit, fertile insecticide resistant insects in early resistance generation studies, no increase in cuticle thickening or selection for P450s which would reduce susceptibility to other insecticides	*Idem*
Relative efficacy against mosquitoes highly resistant to current insecticides	Minimum activity on field *An. arabiensis, An. gambiae* and *An. funestus* via membrane feeding including strains with known insecticide resistance	Activity on all four major *Anopheles* species with malaria relevance in Africa
Drug–drug interactions	No unsurmountable risks with potential anti-malarial partners, especially those under consideration for SMC	No interactions with other anti-malarial, anti-retroviral or tuberculosis medicines
Safety	Safety margin > tenfold between therapeutic exposure and NOAEL in preclinical studies, and easily ‘monitorable’ adverse event or biomarker for human studies.	Safety margin > 50 fold and easily ‘monitorable’ adverse event. No reprotox safety signal in two animal species (‘Minimum’ for MDA, ‘Ideal’ for SMC).
Formulation	Simple and inexpensive to produce, not requiring proprietary methodology or kits; can readily be produced in endemic countries.	Simple and inexpensive to produce, not requiring proprietary methodology or kits; can readily be produced in endemic countries. No food effect.
Cost of active ingredient in final medicine	Similar to current anti-malarials: ≤ $0.5 for adults, $0.1 for infants under 2 years	Similar to older anti-malarials: < $0.25 for adults, $0.05 for infants under 2 years
Estimated stability of final product under Zone IVb conditions (30 °C 75% humidity), in final packaging	≥ 2 years	≥ 3–5 years

TCP-6 criteria for moving a molecule forwards into Phase II

PK, pharmacokinetic; MTD, maximum tolerated dose, NOAEL, no-observed-adverse-effect-level; G6PD, glucose-6-phosphate dehydrogenase

### Use cases: how could an endectocide be deployed for transmission blocking?

Three types of use case can be envisaged. The first is as a stand-alone medicine to be used in MDA, the second as an adjunct to treatment with ACT or the successors, and the third as part of the SMC regimen, again all backed by effective vector control with LLINs or IRS.

Although the ‘stand-alone’ MDA route initially appears to be the most interesting, it is limited since medicines cannot be given to women of unknown pregnancy status in the absence of significant additional clinical safety data. For example, in the case of artemether-lumefantrine, such a compelling data package on safety in the first trimester of pregnancy is only starting to emerge two decades after the launch of that treatment [55]. For new chemical entities, especially those conferring no direct benefit, a more conservative approach is warranted.

The second approach (using in conjunction with ACT) is an option, but currently the majority of infections in Africa are asymptomatic. Unless the guidance on treatment is extended to include asymptomatic infections, then this route will also be limited.

The third approach builds on the success of SMC. In its current implementation, SMC is the use of a full treatment course of sulfadoxine–pyrimethamine and amodiaquine (SP–AQ) to each child aged between 3 and 59 months administered monthly for three or four months throughout the rainy season in areas of highly seasonal malaria. SMC has been enormously successful in reducing the incidence of clinical malaria infection and deaths in the Sahel [56], with over 13 million children protected in 2016. Unfortunately, because of parasite resistance, there are no effective drugs for SMC in areas south of the Sahel [57]; this is a gap which a TCP-6 molecule could fill. Currently, there are discussions to extend the Senegalese programme, to include children up to menarche, a concept known as the ‘Senegalese Ladder’ [4]. Because of the combination of high infection frequency, low immunity and relatively large body surface area children in the age range 6–10 years old are the principal source of transmission [58], with those in the age bracket 5–15 years contributing about the same as all older than 15 years [59]. For example, modelling studies on ivermectin show that with an increased dose (300 µg/kg daily for 3 days, every month throughout the rainy season [49], rather than a single 150 µg/kg dose) a significant additional decrease in incidence of clinical malaria can be obtained when given with SMC up to the age of 10 years (HS, unpublished observation). Broader population coverage is, therefore, clearly expected to have a greater impact on population incidence of infection. However, this requires evidence of safety in first-trimester pregnancy. Thus, a new molecule should be non-teratogenic.

**Fig. 1:**
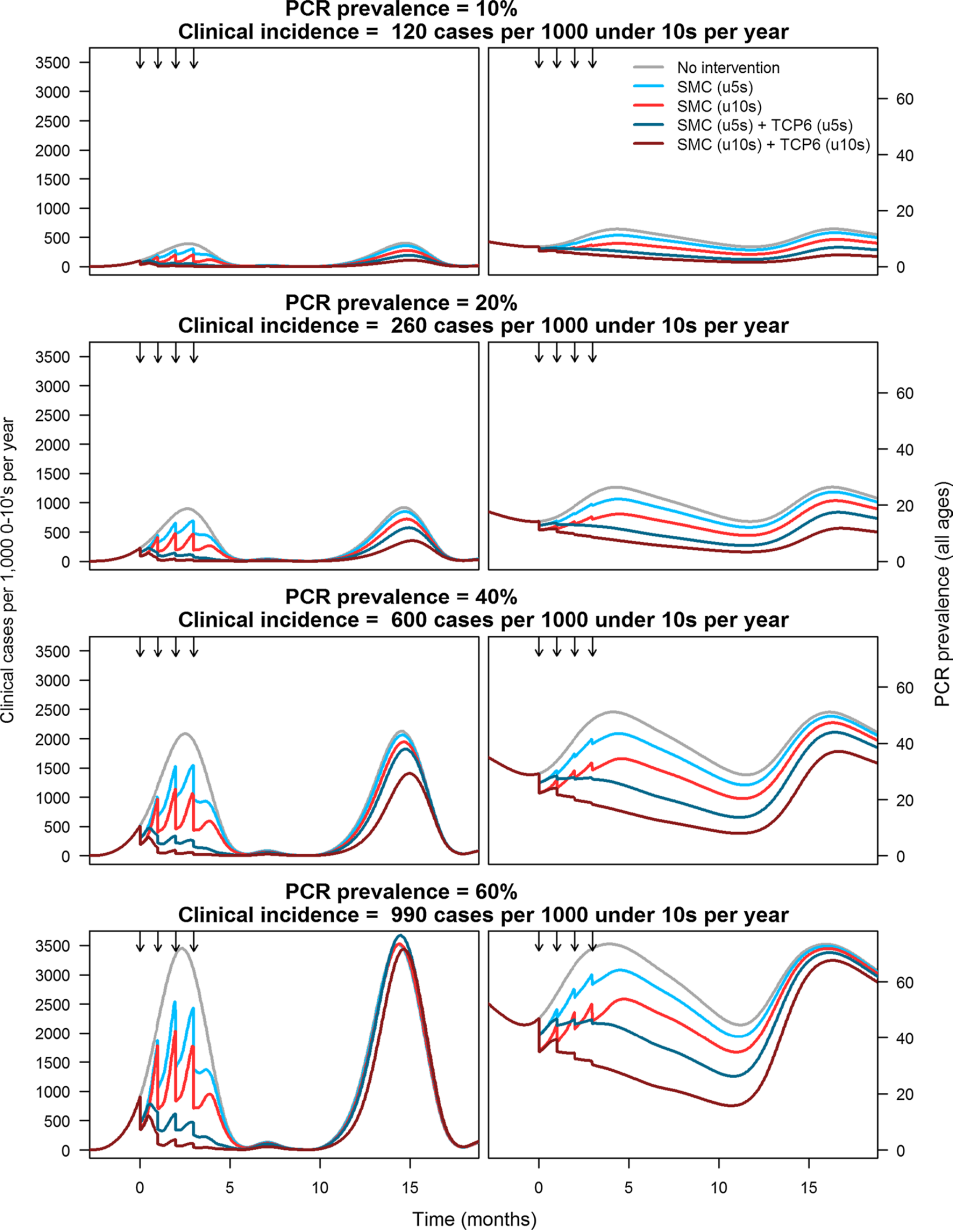
Representative simulations in regions with differing transmission intensities. Four scenarios are considered. In blue, 80% coverage of SMC in under 5 year olds, with no TCP-6 compound and only SP–AQ; in red 80% coverage of SMC under 10 year olds with no TCP-6 compound and only SP–AQ; in dark blue 80% coverage of SMC and a TCP-6 compound in all children under 5; and in magenta 80% coverage of SMC and a TCP-6 compound in all children under 10. The clinical case incidence of symptomatic malaria on the Y-axis for the left-hand figures is the incidence in children 0–10 years of age. The PCR-measured prevalence on the Y-axis for the right-hand figures is in all age groups

Demonstration of efficacy of an endectocide in reducing incidence rates of symptomatic malaria when added to SMC could then be followed by measurements of the impact on asymptomatic malaria, or even parasite prevalence. The initial modelling suggests that treating only children under 10 years of age would provide a significant reduction in mosquito survival and impact parasite prevalence in the wider population (see Fig. 1), and this is proposed as a Phase II study. Demonstration of population efficacy requires larger cluster-randomized studies, which have significant logistical challenges, and so this is reserved for the pivotal Phase III intervention (see below for outlines of the clinical studies).

The deployment of an endectocide would need to be part of a public health agenda, and therefore the high-level customers are the health ministries, the national malaria control programmes and the national vector-borne disease programmes. Therefore, four factors are critically important: a demonstration of safety in the targeted populations, the ease of deployment, a clearly visible early-stage benefit and appropriate cost/benefit, given the scale of deployment necessary. Cost is a critical factor: bearing in mind that SMC costs US$0.30 per month, and treatment with an ACT is less than $0.50 for children, a price target of $0.50 per month has been set, considerably lower than the $1.50–6.00 per day cited in a TCP for ivermectin [46].

### TCP-6 requirements are based on their impact on transmission as predicted by modelling

**Fig. 2:**
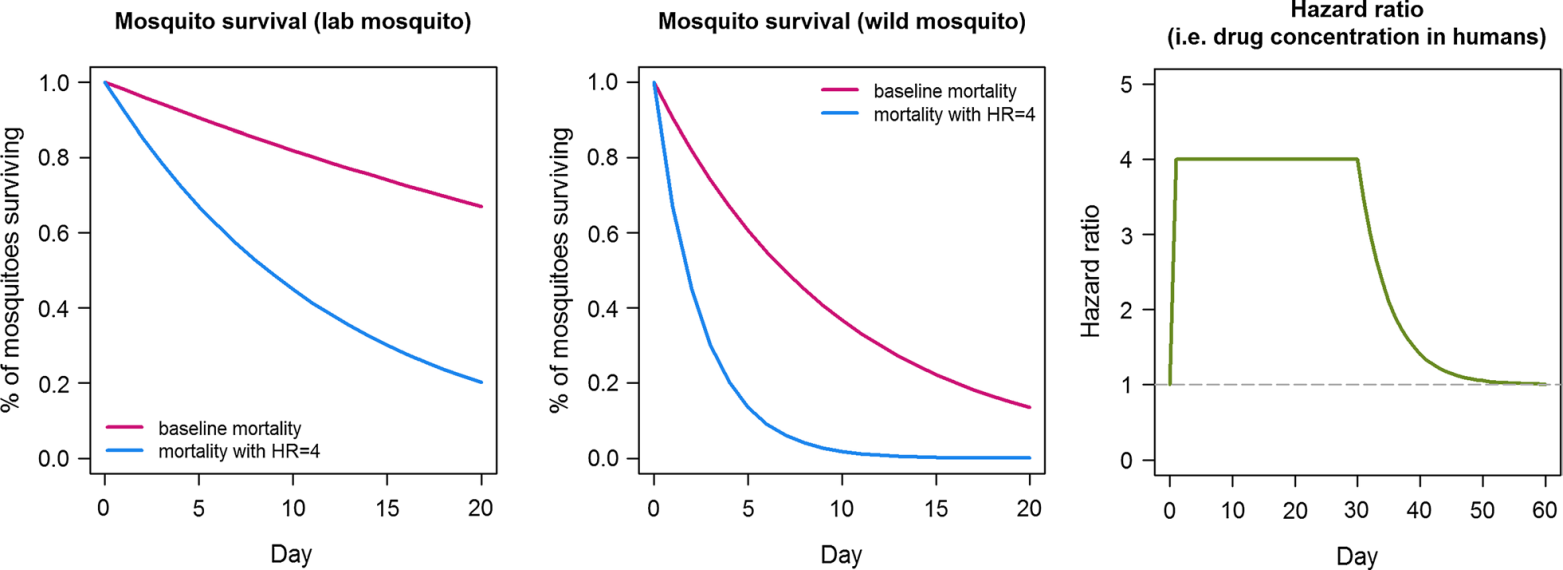
Impact of a TCP-6 compound on mosquito survival, assuming that the compound can maintain a hazard ratio of 4 up until day 30. The model assumes that a mosquito lives for 50 days in the laboratory and for 10 days in the wild. The panel on the right shows the HRs (the ratio of the blue and red lines in the middle and right graphs) plotted over the time expected of the TCP6 lasting in the blood for 30 days

**Fig. 3:**
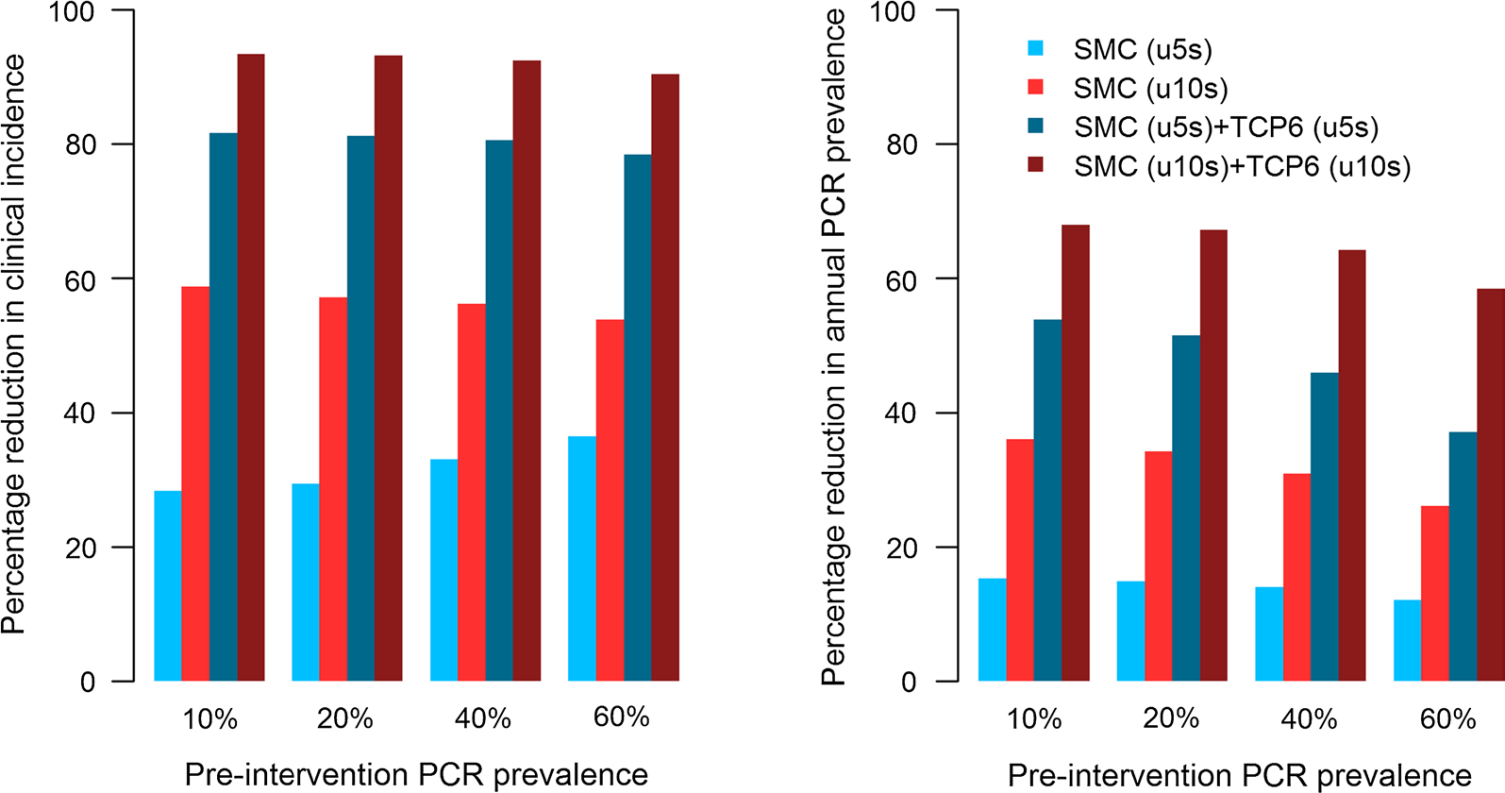
Percentage reduction in clinical incidence of symptomatic malaria in children < 10 years old (left panel) and annual PCR prevalence in all ages (right)

The impact of endectocidal transmission-blocking was assessed using an existing transmission model of malaria [58, 60, 61]. Here the impact of dosing a TCP-6 compound in conjunction with the current gold standard for SMC was measured on the incidence of clinical malaria and parasite prevalence in children 0–10 years of age. These simulations have allowed for the coverage of the population dosed, population characteristics, transmission intensity, and the shape of the hazard ratio-duration curve. Modelling suggests targeting a hazard ratio of 4 at day 30 as appropriate, with simulations suggesting that above this there is minimal additional impact. A hazard ratio of 4 means that a mosquito has a 4 times higher daily probability of mortality compared to a mosquito that has not taken a blood meal containing a TCP-6 compound. In the modelling, a conservative profile is used, where no additional benefit is given for the (early) period where the hazard ratio is above 4, hence the flat appearance of the curve in the first graph of Fig. 2. A coverage of 80% of the intended SMC population along with SMC for four successive months (in children 6 months to 10 years old in regions with parasite prevalence of 7–62%), would deliver substantial reductions in clinical incidence. The observed reductions range between 70 and 90% and 40 and 60%, respectively [49], depending on the transmission intensity category (Fig. 3). Interestingly, clinical studies that examined the ivermectin impact on mosquito survival (when dosed at 3 × 300 μg/kg), based on feeding assays post-dose, have shown that the hazard ratio is 4 at day 14 but only 1.1 at day 30 [49], but even with this inferior profile ivermectin is still predicted to effect a significant reduction in rate of clinical incidence when used in combination with SP–AQ. This suggests that a dose producing a hazard ratio of 4 at day 14 may suffice, reflecting the possibility that the survival times of mosquitoes exposed to the drug may be disproportionally shortened in the wild.

### Protecting against the development of resistance

The mosquito resistance risk against a new TCP-6 molecule needs to be acceptable. WHO guidelines require that for a susceptible mosquito population, new insecticides must deliver 98% mortality within 24 h of contact [62]. Mosquitoes surviving such insecticide contact have an increased risk of developing resistance at the population level. Ivermectin has considerable benefits at concentrations significantly lower than the LC_95_ (the lethal concentration for 95% of the mosquito population) at day 30 post-dosing, due to the decreased fitness and survival of mosquitoes having ingested ivermectin [35]. Determining the precise level of the resistance threat which is acceptable for an endectocidal transmission-blocking strategy will be difficult, but should not discourage development of this product class, especially since emergence and spread of such resistance could be managed by effective combinations with other vector control tools [63]. There is currently insufficient data to determine the degree of fitness reduction from a sub-lethal concentration of different endectocides, though it is likely that such a relationship is mechanism-dependent. Resistance to pyrethroids used in common current vector control interventions is often a result of Cyp P450 overexpression, and does not typically result in a fitness penalty. Thus, a drug targeting TCP-6 should have a different mode of action of resistance to insecticides in ITN and IRS products used in the target communities. This provides an additional safeguard against the emergence and spread of resistance, which may be sufficient to underpin the use of a single endectocide, rather than a combination.

### Safety and tolerability of a new endectocidal transmission-blocking strategy

The new drug may not produce an immediate benefit, in terms of protection from malaria for the individuals enrolled in a treatment campaign, although there will be some effects on other ectoparasites and helminths. Such a medicine would be viewed as altruistic, and the ethical context would be similar to that previously discussed for transmission-blocking vaccines [64, 65]. There are experimental approaches to transmission-blocking vaccines [66, 67], with the most advanced candidate being in clinical testing [68]. The safety and tolerability for a new TCP-6 molecule needs to be at a similar level to that traditionally seen in vaccination programmes. Tolerability is very important here, especially the risk of vomiting, since the compound would be given concomitantly with other medication. This places restrictions on the total mass of the drug, but also underscores that the formulation must be child-friendly. The use in combination with SMC means that the clinical development programme will need to focus on specific drug–drug interactions, especially any that may adversely impact the risk/benefit ratio of the new TCP-6 molecule. Beyond the standard focus on serious and severe adverse events, as in any drug development programme, specific attention will be given to early signal detection for risk of life-threatening adverse events, such as drug-induced anaphylaxis, Stephens-Johnson Syndrome, liver/renal injury, arrhythmias or aplasia. In reality, confidence that such events do not even occur at low frequencies will require continuous pharmacovigilance, and the threshold risk/benefit balance tolerated is likely to be extremely low.

Given that a critical success factor is deployment in as wide an age range as possible, it is important to know if the safety profile potentially allows development for use in pregnancy and children. If no non-clinical signs of developmental and reproductive toxicology are observed in two preclinical mammalian species with a completely new TCP-6 compound, inclusion of women of childbearing age might be permitted in clinical studies. However, a large safety database, most likely from accidental exposure in early pregnancy would be required prior to the use of such an agent in larger population use cases, such as in MDA.

### Approaches to finding new TCP-6 candidates

#### Screening strategies

Screening efforts to find new endectocides have moved from in vivo screens to in vitro screens in the last two decades, highlighted by the early work on avermectins, and more recent work on isoxazolines. In vitro and in vivo screening systems have been developed using membrane feeding assays, known as the ‘artificial dog’ because of their use in rearing fleas for experimental purposes [69]. These have been used to characterize the activity of molecules on commercially important ectoparasites in the absence of a molecular target assay [69]. The artificial membrane system was used to identify and optimize new scaffolds, such as derivatives of the fungal metabolite nodulisporic acid A [70], and characterize over 3000 compounds in the development of the isoxazoline, sarolaner. As with any phenotypic screening programme, this work was eventually supported by data from inhibition of the molecular target in stable cell lines expressing cat flea RDL (resistance-to-dieldrin) genes for assessment of the GABA-gated chloride channel [71].

Primary screening of compounds by the IVCC (Innovative Vector Control Consortium) and partners over the last few years has allowed the assessment of some 4 million compounds as insecticides. However, these have been screened as part of a search for new insecticides for ITNs or IRS, focusing on compounds that are active upon contact with the mosquito. The delivery of an insecticide through oral ingestion provides the potential for the use of other chemistries, including those that do not depend on the uptake through the insect tarsi. Here, the more relevant comparator is the search for new molecules for use in ATSBs [24], since in both cases the molecule is ingested by the mosquito. However, there are still key differences in the properties required: a TCP-6 is required to be orally available in humans and have very high safety and tolerability, whereas an ATSB insecticide just needs to be stable and soluble in the sugar matrix. In practice, the insecticide doses ingested by the insect via these two routes (ATSB or as a TCP-6) will differ vastly, so potency will need to be higher in the latter case.

Another source for potential TCP-6 candidates comes from repurposing advanced compounds from the companion animal endectocide/insecticide portfolio. A recent internal review of the available data by Medicines for Malaria Venture (MMV) and IVCC identified around 200 known insecticides and endectocides; these are currently being assembled into a collection for screening.

#### A process for the characterization and optimization of TCP-6 candidates

**Fig. 4:**
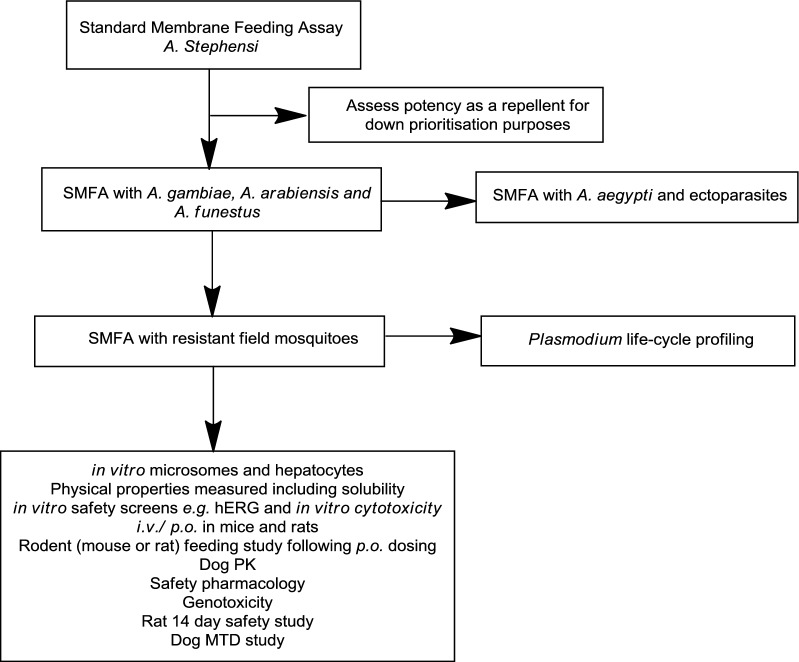
Screening cascade for identifying compounds for TCP-6 or ATSB. The bottom box identifies the suite of studies necessary for full evaluation of a potential TCP-6 candidate drug. If any properties require optimization, then medicinal chemistry would be driven using the *A. stephensi* SMFA to assess potency alongside any of the other relevant non-efficacy assays

The process of screening and compound optimization is described below and in Fig. 4, and is aligned with that described previously for malaria and other neglected diseases [72]. This cascade has a number of critical components:

- The gold standard assay here is the standard membrane-feeding assay (SMFA), in which female *Anopheles* (typically *Anopheles stephensi*) feed on a blood meal in the presence or absence of test compounds. The SMFA is a relatively resource-intensive assay, but recent set-ups that use blood containing DNA barcode-labelled bacteria allows the SMFA to be run in a 96-well format [73]. This assay can be used to compare test compound activity as part of a sugar bait or in a blood meal, to thus establish the relative potential in an ATSB or as a TCP-6. In this assay, the end-point is mortality over time, which can then be expressed statistically using hazard ratios. Once efficacious compounds are identified, a second SMFA could be performed using Stage V gametocyte-infected human blood, to examine concentration effects on subsequent oocyst prevalence. This allows an optional secondary endpoint that explores whether a TCP-6 also blocks transmission at concentrations below that required for mosquitocidal activity.
- The next stage is to demonstrate adequate potency against the main malaria-transmitting *Anopheles* species in Africa: *Anopheles gambiae, Anopheles coluzzii, Anopheles arabiensis,* and *Anopheles funestus*. At this stage, adding broader secondary screening assays including other insect vectors (*Aedes* species) as well as ticks and lice would give valuable information for uses outside malaria.
- Once potency across laboratory species has been confirmed, efficacy against highly insecticide-resistant field mosquitoes is evaluated. The SMFA will need to be adapted to use field mosquitoes. Mosquito resistance is typically due to: up-regulation of P450 s, enhancing toxin metabolism; cuticle thickening, which lowers the permeability of the toxin to the site of action; or mutation of the specific mosquito biological target [74]. It is important to note that an ATSB and TCP-6 with the same mechanism of action or resistance could still be complementary rather than competing tools, when used with each other in accordance with best practice for insecticide resistance management, and other standard measures in a particular region.
- The life cycle fingerprint of such a compound would need to be established, by testing for inhibition against all stages of the *Plasmodium* life cycle. Any activity against the asexual blood or liver stages would be particularly beneficial, since such patient benefit would potentially simplify the clinical development plan.
- Potential TCP-6 agents will also be tested for any repellent effects; this can be done through a choice test between TCP-6-containing blood versus untreated blood. Clearly, repellent activity in such an agent is undesirable.

Beyond these components, the optimization of an endectocide is similar to any other drug, with considerations that the drug will be used primarily as a vector control tool. The focus should therefore be on balancing potency, pharmacokinetics, pharmacodynamics, and safety. The preclinical candidate will need to have appropriate solubility, permeability and pharmacokinetics to give confidence that the predicted human pharmacokinetics in the target population with the proposed dose are sufficient to support monthly dosing. These predictions would initially be based on in vitro data, but supported by in vivo studies in rats and dogs. For an existing insecticide the toxicology part of its regulatory dossier will provide much information about metabolism and clearance of the active ingredient in mammalian systems. Ideally the regimen would consist of a single dose, with a potential for three daily doses in line with current SMC. The requirements for high oral bioavailability and low human clearance place a particular demand for this TCP. The non-clinical safety package is similar to that for any other oral candidate drug [75]. These include off-target pharmacology, ion channel inhibition including hERG (human Ether-a-go-go-Related Gene), in vitro genotoxicity including Ames and micronucleus tests, and phototoxicity studies. The required exploratory non-GLP (Good Laboratory Practice) rat safety study to support a clinical candidate would be a 14-day study, plus at least a 7-day wash-out period to examine reversibility. Because of the need for TCP-6 compounds to be well tolerated, if the biological target in the mosquito is known and orthologues exist in humans, then exquisite selectivity may be required. Finally, a coherent picture of the intellectual property position, including access to existing safety data, will be needed to establish responsibilities moving forwards [76].

### The regulatory strategy for a new TCP-6

#### General considerations

The outline for a TCP-6 clinical development strategy is based on the initial use of the compound as part of a SMC campaign. Use in combination as an add-on to an ACT for MDA or as a tool for malaria control and/or elimination could build from this initial application. Irrespective of the targeted use case, the regulatory registration of the new compound must first be achieved. Any new compound will be developed with a view to obtaining stringent regulatory approval or opinion with the US FDA or EMA (European Medicines Agency) as a first step. This would subsequently lead to WHO-Prequalification and facilitate National Regulatory Authorities approval in malaria-endemic countries.

No drugs are currently approved for such a transmission-blocking strategy; hence no precedent can serve as a basis for the regulatory path. Discussions with regulators as well as the various WHO stakeholders will be essential at the early stages and all along the development to validate the initial concept and overall development plan to achieve registration. Since the current reference, ivermectin, is widely used as a human medicine, this new class, endectocidal transmission-blocking compounds, will likely be regulated through the medicines prequalification pathway, rather than the vector control prequalification route, even if the candidates primarily act on the vectors. The widespread use of ivermectin, and the available safety database may support an abbreviated pathway, but even in this case, there will need to be significant additional data given the increased dose and duration proposed.

Thus, a new indication of ‘transmission-reducing agent’ is proposed. This agent would be added on top of the gold standard SMC regimen SP–AQ as an initial exemplar. SP–AQ was granted WHO-Prequalification, is registered in endemic countries, and is currently recommended by the WHO for use in SMC in children under 5 years of age in the sub-Sahel region. As discussed earlier, there is interest in extending SMC to 5–10 year olds, as is already the case in Senegal, due to the increasing burden of malaria in this age group [4]. This add-on approach could be applicable to other, future approved treatment combinations.

The clinical efficacy endpoints proposed for Phase II and III for a TCP-6 that is added on top of SMC would measure incidence rate reduction in symptomatic malaria, with entomological endpoints being proposed as supportive endpoints. Collecting the secondary entomological endpoint data may also be useful in building evidence of the relationship between entomological and clinical endpoints. This will support the future use of entomological endpoints, ideally as surrogate endpoints, when evaluating TCP-6 on top of MDA with ACT later on.

**Fig. 5:**
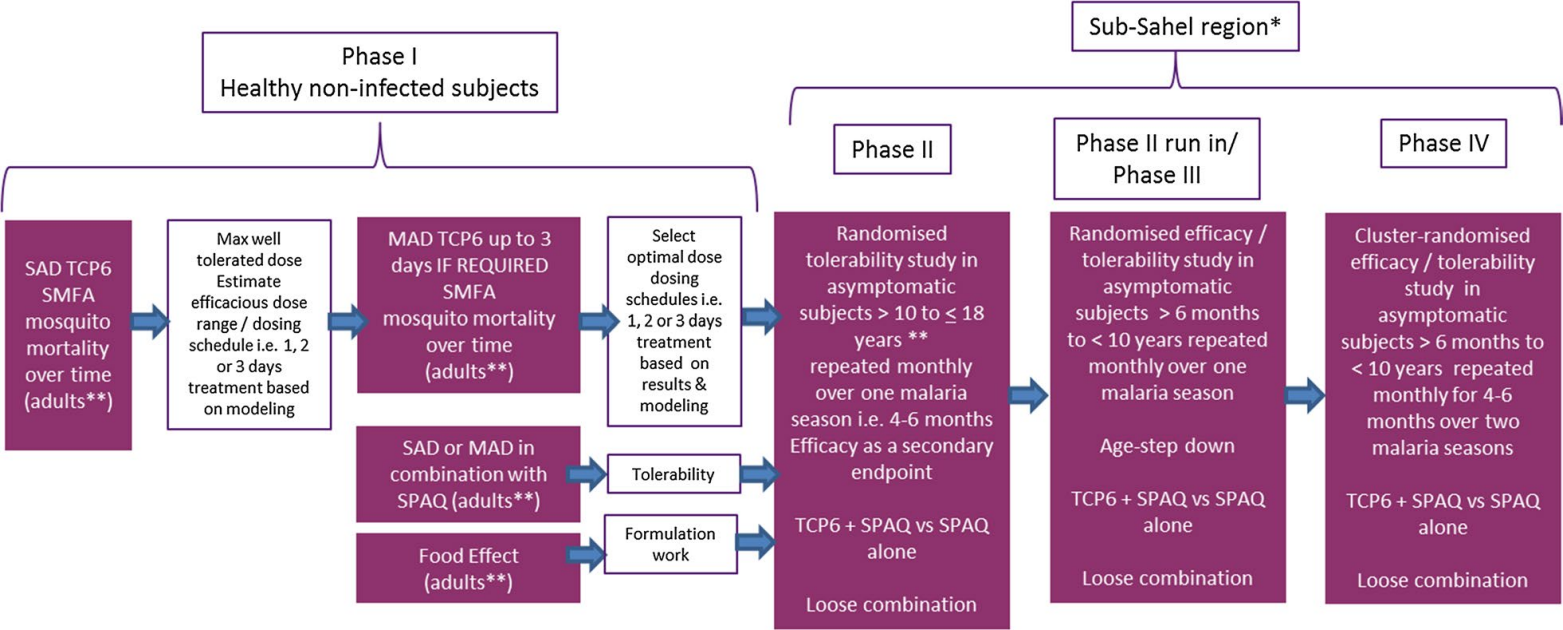
Draft TCP-6 clinical development plan for a novel TCP-6 added to SP–AQ for seasonal malaria chemoprevention in asymptomatic subjects < 10 years of age. *West and Central Africa as required: Burkina Faso, Cameroun, Chad, Gambia, Ghana, Guinea, Guinea Bissau, Niger, Nigeria, Mali, Senegal, Togo; **Excluding women of child-bearing potential; SAD, Single ascending dose study; SMFA, Standard membrane feeding assay; MAD, multiple ascending dose study; SP–AQ, sulfadoxine–pyrimethamine

An outline clinical development plan for a novel TCP-6 added to SP–AQ for SMC in asymptomatic subjects < 10 years of age is proposed in Fig. 5. In the future, additional or alternative SMC combinations will be likely replacements of SP–AQ, though the present discussion focuses on today’s gold standard.

Initial estimates of the relationship between drug concentrations and mosquito mortality will be provided through in vitro assessments, which in conjunction with initial predictions of the human pharmacokinetic profile will guide early clinical development by providing an initial indication of the likely dose range, including whether the target exposure duration is likely to require a 3- or 1-day dosing regimen.

#### Phase I design

In Phase I, the safety and tolerability of the compound will be assessed in parallel with assessments of mosquito mortality using direct feeding or standard membrane feeding of *Anopheles* on adult volunteer blood drawn post-administration. This will enable an assessment of the well-tolerated dose range, which can be further confirmed in safety and tolerability studies of the new compound in combination with SP–AQ in adult healthy volunteers. This will also enable mosquito population modelling, to estimate the compound doses that will exceed a hazard ratio (HR) of 4 for 30 days (to allow co-administration with a SMC that is given every 4 weeks) and thus inform the potential therapeutic dose range to test in Phase II studies (starting in adults, and de-escalating in age to reach the target population). A food-effect study will also be required, since, optimally, there should be no food effect for this drug. In parallel with this Phase I study, it would be important to start the early embryo fetal development studies in rats and rabbits and other standard genotoxic and safety pharmacology studies. In the event of a safety signal, this would down-prioritize the use of such a molecule in women of unknown pregnancy status, and thus affect reaching the longer-term MDA goal, although it would not prevent continued development for SMC. Given the use in combination, drug–drug interaction studies may be necessary with any drugs for use in SMC, based on the outcome of SimCyp simulations [77] and in vitro drug–drug interaction assessments.

#### Phase II and Phase III study designs

Phase II and Phase III studies will be carried out in the sub-Sahel regions, in which SMC with SP–AQ is well established in children who are asymptomatic, and hence may be either parasite-free or have sub-clinical *P. falciparum* infection (symptomatic patients are treated with the local standard of care anti-malarial combination). The objectives of these studies are to confirm good safety or tolerability by cautious age de-escalation, to reach the target extended SMC population of 5–10 years, and eventually, if possible, to descend to the 6 months–5 years range. The ultimate goal is to demonstrate superior efficacy of TCP-6 plus SP–AQ compared with SP–AQ alone (in Phase III).

The primary efficacy endpoint will be the cumulative incidence rate reduction (IRR) of symptomatic malaria cases, however secondary endpoints, such as asexual parasite prevalence and additional supportive entomological endpoints could include, for example: parity rate, mosquito density, or the 3-day survival of caught mosquitoes.

Depending on the interactions with the Stringent Regulatory Authorities, this package up to Phase III could potentially lead to registration. A proposed Phase IV study will be a cluster randomized study to demonstrate, as for Phase III, statistical superior IRR of symptomatic malaria cases of TCP-6 plus SP–AQ versus SP–AQ alone, and also to measure an effect on the total human population, rather than just those treated on population prevalence of symptomatic malaria cases, positive parasitaemia and gametocytes, to demonstrate an impact on transmission. Theoretically, the population evaluated in this study could be expanded to subjects > 10 years of age.

## Conclusions

The continued drive towards malaria elimination requires a combination of better and more extensive deployment of existing tools, and the development of new ones. One area that has been discussed frequently is the specific application of transmission-reduction tools. Much of the focus in the past has been on vector control through ITNs and IRS, transmission-blocking vaccines, or the deployment of single, low-dose primaquine. The additional approach of using an endectocide to deliver a lethal dose of drug to the insect has received less attention, but this is changing with recent work on avermectins and isoxazolines.

Whether or not ivermectin is ultimately deployed, there is still a role for new compounds, either improving on safety, convenience and ease of use, or to help target emerging resistance in the insect vectors. To help conceptualize this, a TCP for a new chemical entity targeting the insect vector has been developed here: TCP-6. This lays out the key issues in terms of drug discovery and lead optimization, knowing that the work here is at the interface of traditional drug discovery and vector control. Using this framework, a screening cascade was developed which can help support a logical progression of compounds towards a clinical candidate.

The clinical development pathway for such a transmission-reducing agent is complicated, in that there is no precedent, and hence a constant dialogue with the regulatory authorities and the WHO will be essential. Several use cases have been established, but the addition of a new endectocide to SMC has a certain attraction as an initial approach. First, it does not require the early generation of evidence for safety in the first, second or third trimester of pregnancy. Second, although the modelling suggests that as large a population as possible should be targeted, even targeting those up to the ages of 5 and 10 is predicted to produce a significant decrease in the incidence and prevalence of infection. Modelling also suggests that dosing and human pharmacokinetics must produce a HR of 4 that is maintained up to 30 days.

This definition comes at an important time. The last few years of vector control have been focused on insecticides that work for LLINs, and thus are effective because of direct physical contact. Recently there has been an increased interest in ATSBs that deliver the insecticide to the mosquito by ingestion. The overlap between the requirements for a compound active as an ATSB and one active for endectocide transmission blocking are significant. This synergy gives renewed optimism that such compounds can be identified and developed.

## Abbreviations

ACT: artemisinin-based combination therapy
ATSB: attractive targeted sugar bait
BFI: blood-feeding inhibition
EMA: European Medicines Agency
FDA: Food and Drug Administration
G6PD: glucose-6-phosphate dehydrogenase
GLP: Good Laboratory Practice
hERG: Human ether-a-go-go-related gene
HR: hazard ratio
IPTp: intermittent preventive treatment in pregnancy
IRR: incident rate reduction
IRS: indoor residual spraying
ITN: insecticide-treated net
IVCC: Innovative Vector Control Consortium
LLIN: long-lasting insecticidal net
MAD: multiple ascending dose study
MDA: mass drug administration
MMV: Medicines for Malaria Venture
MTD: maximum tolerated dose
NOAEL: no-observed-adverse-effect-level
PCR: polymerase chain reaction
PK: pharmacokinetic
POC: proof of concept
SAD: single ascending dose study
SMC: seasonal malaria chemoprevention
SMFA: standard membrane feeding assay
SP–AQ: sulfadoxine–pyrimethamine plus amodiaquine
TCP: target candidate profile
TPP: target product profile
WHO: World Health Organization
